# The perfect personalized cancer therapy: cancer vaccines against neoantigens

**DOI:** 10.1186/s13046-018-0751-1

**Published:** 2018-04-20

**Authors:** Luigi Aurisicchio, Matteo Pallocca, Gennaro Ciliberto, Fabio Palombo

**Affiliations:** 1 Takis, Rome, Italy; 2 Biogem, Ariano Irpino, Italy; 3 UOSD SAFU, IRCSS Regina Elena National Cancer Institute, Rome, Italy; 4 Scientific Directorate, IRCCS Regina Elena National Cancer Institute, Rome, Italy; 5 Alleanza contro il Cancro, Rome, Italy

## Abstract

In the advent of Immune Checkpoint inhibitors (ICI) and of CAR-T adoptive T-cells, the new frontier in Oncology is Cancer Immunotherapy because of its ability to provide long term clinical benefit in metastatic disease in several solid and liquid tumor types. It is now clear that ICI acts by unmasking preexisting immune responses as well as by inducing de novo responses against tumor neoantigens. Thanks to theprogress made in genomics technologies and the evolution of bioinformatics, neoantigens represent ideal targets, due to their specific expression in cancer tissue and the potential lack of side effects. In this review, we discuss the promise of preclinical and clinical results with mutation-derived neoantigen cancer vaccines (NCVs) along with the current limitations from bioinformatics prediction to manufacturing an effective new therapeutic approach.

## Background

Even though cancer therapy has made significant advances in the last decade, in the majority of cases it still fails to achieve long-lasting responses in patients with metastatic disease. To explain the reasons why tumors relapse the clonal evolution model has been proposed to reveal how intra-tumor heterogeneity (TH) is the basis for emerging tumor variants under targeted therapies and immunological pressures [1].

The use of next generation sequencing (NGS) for massive analysis of cancer genomes allows a quantitative measurement of mutational frequencies and genome copy variations. The cancer atlas is quite diverse, ranging from a few to thousands of mutations for individual histological tumors [2], thus raising concerns on how to deal with this high complexity. Mutations are classified according to their role in tumor growth. Most of them do not confer intrinsic growth advantage and are defined “*passenger mutations*” whereas a smaller number of them, known as “*driver mutations*”, provide a growth advantage and are therefore selected during tumor evolution. Druggable mutations, a subset of driver mutations, are defined by the availability of a drug (or the possibility to generate a drug) capable of targeting a specific genomic alteration. An intense research activity has currently been launched towards extending the use of such drugs to most tumor types which carry a selected mutation. Most of these mutations encode amino acid substitutions and therefore are collectively known as nonsynonymous mutations, resulting in new, cancer-specific protein sequence not expressed in normal tissues.

The analysis of different regions of the same tumor revealed that some mutations are commonly present (clonal) while others are unique only in some parts of it (subclonal) contributing to TH. High TH may explain why initial clinical responses defined by the reduction of tumor mass can fail at later times due to the outgrowth by treatment-resistant cancer subpopulations. It is important to stress that under selective pressures, tumor evolution can be redirected according to the timing and type of cancer therapy [3]. Ideally, we need to combine therapies against as many possible tumor-specific targets in order to reduce the likelihood of emerging escape variants. Small molecule inhibitors as well as biologics raised against driver/actionable mutations are designed against one target at the time, requiring a long development process, which results in a limited available armamentarium with a series of related side-effects. In this scenario, the feasibility of a multivalent target therapy made of small molecules or biologics is limited by practical reasons and cumulative side-effects associated with therapeutic drugs.

The promise of a personalized cancer vaccine is therefore to target multiple tumor specific mutations reducing side-effects by sparing normal tissue and keeping tumors under immunological memory control for as long as possible. In this review, we describe the mechanisms underlying the basis of immune recognition of tumor cells and the evidence of preclinical and clinical studies in the emerging field of mutation-derived neoantigen cancer vaccines.

## T-cell immune response against self- and non-self antigens

T-cells are capable to recognize and kill cells presenting on their surface non-self or altered self-antigens, i.e. peptides derived from intracellular protein cleavage. Proteins are cleaved by the proteasome generating a peptide pool, which is loaded into the endoplasmic reticulum by the TAP-1 system. In order to be presented on the cell surface, peptides are further trimmed and complexed with major histocompatibility complex (MHC; also known as human leukocyte antigen - HLA - in humans) class I molecules for their presentation to CD8+ T cells. MHC-I is a heterodimer composed of a polymorphic heavy chain and β2-microglobulin. Peptides are also presented by MHC-class II molecules when they are digested through autophagy. MHC-II complexes are exposed to the immune system by antigen presenting cells (APC), such as dendritic cells (DC), and upon IFN-γ stimulation also by other cell types including epithelial cells [4]. MHC-II presented peptides derived from proteins digested in the endocytic pathway are recognized by CD4+ T-cells. The subset of peptides capable of stimulating T-cells are defined antigens.

Decades of research have led to the identification of a large number of self tumor antigens derived from the processing of normal proteins that have been grouped into three categories: tumor associated antigens (TAAs), tumor specific antigens (TSAs) and cancer testis antigens (CTAs). TAAs are defined as those antigens overexpressed by cancer cells than normal tissues. TSAs are those specifically expressed only in cancer cells and not in normal tissues. CTAs are expressed, besides tumor cells, only in germline tissues and trophoblastic cells [5]. These antigens have been the focus of intense pre-clinical and clinical research in the attempt to generate therapeutic cancer vaccines targeting these antigens. Unfortunately, in spite of encouraging pre-clinical data, a lifetime worth of clinical cancer research with these antigens has led to the conclusion that breaking immunological tolerance against self-antigens is actually more difficult than originally anticipated. In the meantime, the massive use of “omics” in cancer research has revealed that non-self-antigens derived from non-synonymous mutations in the coding region of proteins are instead efficiently recognized by the T-cell specific immune response (reviewed in [5, 6]). In this review, we will not discuss antigens derived from post translational modifications as it has recently been published in a paper [7] but only mutation-derived ones that we will refer to as neoantigens.

Several lines of evidence support neoantigens as being important targets for immune responses. A higher neoantigen load was indeed associated with improved patient survival in a study that assessed hundreds of tumors with 6 different histological types from the TCGA [8]. An association between neoantigen load, increased number of tumor infiltrating lymphocytes (TILs) and improved survival was observed in colorectal [9] and endometrial cancer [10]. Neoantigen-specific T-cell immunity correlates with clinical response to immune checkpoint inhibitors (ICI) [11].

Monoclonal antibodies interfering with the programmed cell death protein 1 (PD1) and cytotoxic T lymphocyte antigen 4 (CTLA-4) signaling pathway are effective in many solid and hematological malignancies leading the FDA to approve their use in a growing list of tumors with different types of histology [12]. The clinical response to ICI treatment indeed correlates with neoantigen load in patients with melanoma [13], non-small-cell lung cancer (NSCLC) [14], and colorectal cancer [15]. Moreover, neoantigen-specific T-cell responses become evident in patients treated with ipilimumab (anti-CTLA-4) and with pembrolizumab (anti PD1). Although high neoantigen load is associated with good prognosis, the nature of tumor mutations is also relevant for the therapy based on ICI [16]. High levels of TH is associated with resistance and tumor escape [16]. A possible explanation of this may be the limited number of responses against neoantigens observed in patients treated with ICI as compared to the neoantigen repertoire presented by tumor cells [17]. Finally, in a separate set of observations with adoptive T-cell transfer, patients with solid tumors showed measurable T-cell specific immune responses against neoantigens [18, 19]. On this basis, neoantigen cancer vaccines (NCVs) may represent an emerging new clinical approach to treat cancer.

## NCVs in preclinical tumor models

**Fig. 1:**
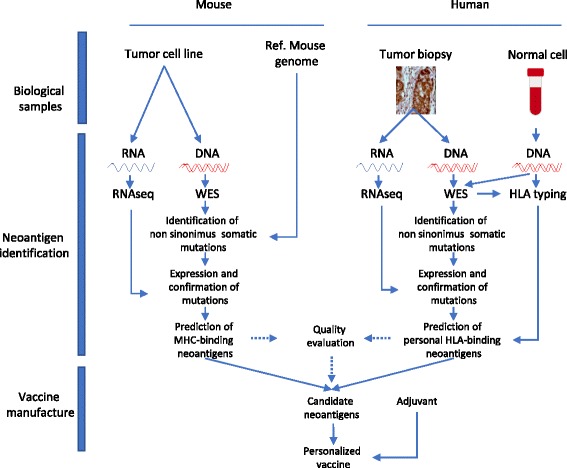
The pipeline of neoantigen cancer vaccine production, for mouse studies (left side and human studies (right side). 1. Tumor and normal tissue are collected and subjected to (2) exome sequencing and RNAseq analysis for the tumor samples. 3. expressed non-synonymous mutations are then further selected according to binding to predictive algorithms and incorporated in a vaccine vector or delivered as peptides with adjuvants

NCVs have proven to be effective in different preclinical animal models (Table 1). The current method used to identify neoantigens and generate NCVs [20] is based on the following three steps (Fig. 1): 1) Collection of tumor and normal samples; 2) identification of neoantigens; 3) formulation of the vaccine. In the mouse system, non-synonymous tumor-specific point mutations are identified by comparison of exome sequencing data of the tumor cell line of interest with reference to the mouse genome. In order to be immunogenic, a neoantigen has to be expressed. Therefore mutations are further selected according to the level of gene expression measured by RNA-seq. Finally, the expressed neoantigens are ranked according to different bioinformatic pipelines as described below. The most popular methods to predict binding to MHC are NetMHC-4 and NetMHCpan [21]. The last step is the delivery of neoantigens in an immunogenic formulation that includes peptides complexed with adjuvants [20] or with liposome particles [22]or delivered as an RNA vaccine [22]. This workflow results in cancer-specific immune responses that are efficacious against several tumor types including melanoma, colon cancer and sarcoma (Table 1). The pipeline for NCV production in preclinical mouse models can be further refined by the introduction of immunoproteomic methods designed to discover neoantigens associated with MHC-I complex as it was shown in a colon cancer model [23]. The validity of the neoantigens identified by this approach was further supported by the confirmation of the immune responses in a subsequent work where the neoantigens were successfully utilized with a different vaccination platform [24].

**Table 1:** Preclinical data with NCV

Type of neoantigen vaccine	Formulation	Type of Tumor	Checkpoint Inhibitor Blocked	Anti tumor effects	Ref
peptides	poly(I:C)	melanoma	–	tumor delay	[20]
peptides	anti-CD40 antibody + poly(I:C)	Colon and prostate	–	tumor delay	[23]
peptides	–	Fibrosarcoma	–	tumor delay	[25]
peptides	poly(I:C)	Sarcoma	anti PD-1	tumor delay	[63]
peptides	poly(I:C)	sarcoma	–	tumor delay	[27]
RNA and peptides	RNA complexed with cationic lipids	Colon, melanoma, mammary carcinoma	–	tumor delay	[22]
RNA	RNA complexed with cationic lipids	Colon	–	tumor delay	[28]
peptides	poly(I:C)	ovary	–	No efficacy	[30]
peptides	lipoprotein-mimicking nanodiscs	colon, melanoma	anti PD-1	tumor delay and tumor eradication	[29]
Long peptides	poly(I:C)	Head and neck cancer	–	tumor delay	[31]

NCVs-induced immune responses are in most cases specific for the neoantigens. The initial study provided evidence of responses with some cross-reactivity to wild type cognate epitopes measured by ELIspot assay [20]. On the contrary, subsequent papers showed a more stringent specificity for neoantigens likely due to the use of shorter peptides for flow cytometry analysis and the employment of dextramer staining for the detection of neoantigen-specific T-cells [23–27]. The most surprising evidence emerging from mouse studies is the observation that NCV induces not only a CD8+ but also a CD4 + T-cell response, and that the CD4^+^ T-cell response is primarily responsible for the therapeutic effects [22]. This observation has initially been described using an innovative RNA vaccination platform [28] and was later confirmed by an independent group, which utilized a vaccine based on peptides [29]. Only one study combined NCV with anti-PD1 treatment [29]. This study suggests an additive effect of NCVs and immunotherapy on tumor growth inhibition. Notably, one report did not show antitumor activity in an ovarian cancer model despite the induction of a significant T-cell specific response against neoantigens [30]. The authors highlighted the limited number of mutations in this tumor type and the lack of high-affinity neoantigens, which may be detrimental for an effective NCV approach. A recent paper explored a head and neck cancer model providing further evidence that NCV is able to prevent tumor growth [31].

Whilst these initial studies describing different vaccination platforms and detection systems consistently support NCVs as a promising approach, some questions still remain unanswered. The first is that it is not clear whether the same neoantigen sequences are equally effective using different vaccination methods. It is worth mentioning that prediction of immunogenicity is mostly based on peptide vaccines that may be not informative for other vaccination platforms. Immunodominant epitopes may rank differently or even may not be confirmed in a context dependent manner. Our experience and observations from other groups in the field suggests that further research is needed to determine how vaccination technologies impact the quality of the immune response. It would be useful to generate a comprehensive neoantigen database that takes into account all the steps for the NCVs process including the delivery method and the resulting immune responses in order to improve prediction models. A second question concerns the potential cross-reactivity of neoantigens with wild-type sequences. In this case immunological potency may be limited by central and peripheral tolerance leading to an ineffective T-cell response against the tumor. This class of neoantigens may, therefore, be more similar to the classical TAAs and may result in lower immunogenicity. In addition, vaccination with this group of neoantigens may cause potential side-effects against normal tissues, particularly when a vaccine could contain several cross-reacting neoantigens, which can lead to cumulative side-effects. To be on the safe side we suggest to exclude them from the design of a NCV.

## NCVs in clinical trials

The efficacy of targeting tumor-specific non-self-antigens has been demonstrated in the case of cervical cancer driven by HPV [32, 33]. The immunogenicity of HPV is well documented by prophylactic HPV vaccines, proven to be effective in preventing cervical cancer in young adolescents. For the therapeutic approach the vaccine has to target a different group of viral proteins, namely the oncogenic E6 and E7. A plasmid DNA encoding HPV oncogenic proteins was administered in conjunction with electroporation as the delivery method to induce CD8+ effector T-cells. Targeting key viral proteins E6/E7 resulted in reduction or stabilization of cervical intraepithelial neoplasia (CIN) 2/3 in 50% of patients [33] and in specific immune responses against the HPV targets [34]. In contrast, a similar vaccine technology delivering a fusion protein made of a self-TAA fused to an immunogenic bacterial antigen resulted in immune responses measured only against the non-self portion of the antigen, further supporting the idea that non-self antigens are immunogenic even in potentially immunocompromised patients with high tumor burden [35].

The design of mutation-derived tumor-specific NCVs in human clinical trials recapitulates the mouse protocol with some additional steps (Fig. 1). Tumor biopsy analyses are in fact much more complex than cancer cell lines (as reported in mouse studies) and in most cases the use of formalin fixed paraffin embedded slices as a source material is a factor for a good quality RNA-seq. For some tumors, the low amount of tumor material requires an increased sequencing depth to reveal the presence of rare cancer mutations. Reference normal tissue, usually available as blood samples, serves not only to compare tumor genome with the aim to identify somatic mutations but also to establish the individual HLA. The highly polymorphic nature of the HLA locus poses an issue for the prediction of neoantigens, since limited information is available for rare HLA. Although the prediction pipeline requires additional bioinformatic work, many tools are already available on the web and moreover clinical trials with cancer-specific neoantigens have been reported in melanoma patients using different vaccination strategies [36–38]. Three HLA-A2.1 positive melanoma patients, who had been pretreated with ipilimumab, were vaccinated with DC loaded with peptides encompassing the neoantigen mutations (NCT00683670) [36]. Predicted neoantigens were further selected according to a binding assay using HLA-A2.1 expressing T2 cells and seven validated peptides were used for each patient. Immune responses were detected in all patients although the assay required an in vitro growth of T cells with IL-2. The vaccine expanded T-cells against preexisting dominant epitopes and induced new responses, which were absent before treatment. More recently, a second clinical trial with peptide vaccines has been reported (NCT01970358) [37]. Six naive melanoma patients were vaccinated with a pool of synthetic long peptides + adjuvant. Up to 20 neoantigens were injected in 4 different sites upon formulation with poly-dIdC. Overall, the authors confirmed specific T-cell responses for 24 out of 28 neoantigens. Most of the responses were mediated by CD4^+^ T-cells, however none of the neoantigen-specific T-cells recognized cultured tumor cells in four out of six patients. The two patients with stage IV M1b relapsed after the last vaccination and were treated with anti-PD1. Both showed a clinical response, although the response rate in this subgroup of patients treated with ICI is expected to be only 61%. Upon ICI treatment, new CD4 and CD8 responses against neoantigens were observed. In a third study (NCT02035956) [38], the vaccination with RNA was effective in inducing strong neoantigen-specific CD4 and CD8 responses in 13 melanoma patients in line with previous mouse evidence from the same research group [22]. Eight patients remained tumor free for the follow up period (12/24 months) whereas five patients relapsed during immune therapy. One patient was treated with a combination of NCV and ICI, with a good response. A second patient did not respond to NCVs/ICI and died. In this patient, the analysis of recurring metastasis showed the biallelic loss of β2 microglobulin as the explanation for the lack of tumor response. The predominant CD4 response was also evident for the RNA based vaccination in addition to a relevant percentage of promiscuous double positive CD4 and CD8 neoantigens.

These results taken together suggest that NCVs may turn out to be a suitable clinical approach for highly heterogeneous tumors providing the best balance/ratio between targeting tumors (specificity) while sparing normal tissue (toxicity). However, confirmatory data in larger studies are needed to confirm. Indeed, several active clinical trials with NCVs are ongoing (see https://clinicaltrials.gov/) with different vaccination technologies and targeting different cancers. The most common are basket trial targeting different tumor histology (NCT02992977, NCT03289962, NCT02897765) or lung cancer (NCT02956551, NCT03380871, NCT03166254), followed by glioblastoma (NCT03422094, NCT02287428) and disease-specific trials (Table 2).

**Table 2:** NCV studies in clinical development trial

Clinical trial	Cancer	Intervention	vaccine	status
NCT01885702	Colorectal Cancer	DC vaccination	DC	Active, not recruiting
NCT02632019	Biliary tract tumor	DC vaccination and gemcitabine	DC	Unknown status
NCT02956551	Lung cancer	DC vaccination	DC	Not yet recruiting
NCT03122106	Pancreatic Cancer	Neoantigen DNA vaccine with electroporation	DNA	Recruiting
NCT03199040	Triple Negative Breast Cancer	Neoantigen DNA vaccine and Durvalumab	DNA	Not yet recruiting
NCT01970358	Melanoma	Peptides with Poly-ICLC	peptide	Active, not recruiting
NCT03068832	Pediatric Brain Tumor	Peptide vaccine with Poly ICLC	peptide	Not yet recruiting
NCT02287428	Glioblastoma	NeoAntigen Vaccine and Radiation Therapy	peptides	Active, not recruiting
NCT02897765	Many	NEO-PV-01 and Nivolumab	peptides	Recruiting
NCT02950766	Kidney Cancer	Drug: NeoVax|Drug: Ipilimumab	peptides	Not yet recruiting
NCT03166254	Lung Cancer	Long peptide with Poly ICLC and Pembrolizumab	Peptides	Not yet recruiting
NCT03219450	Lymphocytic Leukemia	NeoVax with Cyclophosphamide	peptides	Not yet recruiting
NCT03359239	Urothelial Bladder Cancer	Peptides with Poly ICLC and Atezolizumab	peptides	Not yet recruiting
NCT03361852	Follicular Lymphoma	Neo Vax and Rituximab	peptides	Not yet recruiting
NCT03380871	Lung Cancer	NEO-PV-01 Pembrolizumab Carboplatin Pemetrexed	peptides	Not yet recruiting
NCT03422094	Glioblastoma	NeoVax and Nivolumab Ipilimumab	Peptides	Not yet recruiting
NCT03289962	Many	Drug: RO7198457|Drug: Atezolizumab	RNA	Recruiting
NCT02992977	many	AutoSynVax TM vaccine	HSP Peptides	Active, not recruiting

## Bioinformatic methods for neoantigen prediction

One of the main issues for NCVs development is the correct prediction of neoantigens. Several bioinformatic tools have been designed in order to call putative neoantigens from genomic data (https://www.ncbi.nlm.nih.gov/pubmed/27376489). The increasing interest in this matter is proven by the fact that 5 out of 7 publicly available pipelines were presented last year.

Neoantigen prediction involves a series of computational steps that can be inferred with specific experimental techniques (Fig. 1). It is for this reason, that bioinformaticians in previous years have focused on creating specialized software for specific sub-tasks (e.g. HLA typing from sequences as well as allele specific expression tools, [39–42], or fit-for-all environments with complex pipelines that address several, or even all, analytical tasks (Table 3). Table 3 contains packages that are meant to be “plug and play” even if the installation process of such a framework can be cumbersome. Furthermore, to our knowledge, there is no freely and publicly available cloud web tool able to process all the required steps for neoantigen prediction from genomic data (https://www.ncbi.nlm.nih.gov/pubmed/27376489).

**Table 3:** Pipelines for neoantigen prediction

Title	Input	Notes	Date	Ref
TIminer: NGS data mining pipeline for cancer mmunology and immunotherapy.	RNA-seq BAM and VCF	Computes GSEA and IPS	10/2017	[64]
CloudNeo: a cloud pipeline for identifying patient-specific tumor neoantigens.	BAM for HLA and VCF	Computes HLA type and Neoantigens	10/2017	[65]
TSNAD: an integrated software for cancer somatic mutation and tumour-specific neoantigen detection.	FASTQ; BAM for HLA	Neoantigen detection pipeline	05/2017	[66]
INTEGRATE-neo: a pipeline for personalized gene fusion neoantigen discovery.	FASTQ	Gene fusion prediction and neoantigen computation from gene fusions	02/2017	[67]
pVAC-Seq: A genome-guided in silico approach to identifying tumor neoantigens.	prepare FASTA (prepare input) and predicts neoantigens	Neoantigen calling, HLA typing, MHC binding	01/2016	[68]
neoantigenR: An annotation based pipeline for tumor neoantigen identification from sequencing data	GSS + FASTA	R package, uses MHC, unpublished		[43]

The typical steps of a neoantigen extraction method starts with the computation of allele-specific coverage. The algorithms typically use aligned sequence data from total RNA-seq and a list of variants from exome/genome sequencing to infer the relative wild type/mutant expression levels at base/mutation level of resolution. With this output it is possible to compute the mutated protein sequence through dedicated software for the assignment of the mutation to the correct protein. The predicted epitopes are then processed with prediction methods that rank the epitopes for binding affinity. This simple three step process (allele coverage/sequence translation/binding prediction) contains several caveats that can hinder the whole process by calling false positives (non-existent epitopes) or false negatives (missed epitopes).

In the translation process, it is obviously of utter importance to choose the right transcript isoform to translate. This step is not so obvious when the mutant allele coverage is computed at the base level, i.e. it is required to understand which of the overlapping expressed isoforms harbor that mutation. If the computation of the exact transcript results to be a process too cumbersome, a decent tradeoff is to choose the dominant transcript for the putative neoantigen identification since it has been shown that most highly expressed genes have one dominant isoform [43].

Another issue related to transcript identification is the relative abundance of expression, inferable from the normalized coverage, since a reasonable choice would be not to include epitopes that are poorly expressed. The threshold for “low abundance expression” is a matter of discussion in the bioinformatic community involved in RNA-seq data analysis. Since an expression level of FPKM (Fragments Per Kilobase of transcript per Million mapped reads) between 1 and 5 represents around 1 transcript copy per cell, the most reasonable way of proceeding would be to eliminate all epitopes generating from isoforms of FPKM < 5. At the base level, since there is no accepted threshold for the RPM expression level of the mutation itself. Hence, one possibility may be to adhere to the transcript FPKM filter and to a high relative MUT/WT ratio.

The authors themselves have implemented a simple method called NaRciSo, in order to extract a list of expressed epitopes from paired Exome and RNA-seq data or standalone RNA-seq (manuscript in preparation). One of its modules is meant to predict neoantigens in the absence of exome sequencing data, computing a “RNA VCF” from RNA-seq sequence data and fetching it to the allele counter package.

Finally, to our knowledge the current available prediction tools that process from sequence reads to neoantigen calls do not try to compute the probability of trimming from ERAP1 (proteosomal cleavage) and peptide processing from TAP1/TAP2, even if some modeling work has been done in the past [44–47]. A few notable exceptions do exist but they start the analysis from preprocessed FASTA files, such as NetTepi (https://www.ncbi.nlm.nih.gov/pubmed/24863339) and NetCTL (https://www.ncbi.nlm.nih.gov/pubmed/20379710), including also a prediction method for T cell reactivity. It is reasonable to think that the integration of these additional modules would increase the prediction power in terms of specificity. 

The effective prediction of immunogenicity can benefit from some additional modeling on the quality of the neoantigen. In this context, an initial hypothesis was formulated in mice where effective neoantigen vaccines were based on a neoantigen with higher binding affinity than the corresponding WT epitope as a means of predicting NetMHC [25]. This feature may spare neoantigens (somatic mutations) from immunological tolerance, which deletes self-reactive T-cells centrally or in periphery. Several papers have explored the immune responses against neoantigens in patients treated with ICI endowed with defined features that better correlate with clinical outcomes. Common sequence motifs similar/homolog to viral epitopes were identified in neoantigens correlating with good prognosis [48]. In accordance with this hypothesis, two bioinformatic papers proposed a “neoantigen fitness model” in order to rank and select the dominant clone-specific neoantigen [49, 50]. This fitness model is computed by taking into account two main factors: the probability of MHC presentation and T-cell recognition. The first factor is derived from the neoantigen binding affinity, with a matched wild type smoothing factor, as there is indeed a minimal “distance” required from the wild type counterpart. The second factor is computed from the neoantigen similarity with a database of known epitopes. Striking experimental evidence showed effective immunological response against the predicted neoantigens and their viral homolog but not against the neoantigen corresponding self-peptide [49]. These data strongly suggest that quality of neoantigens may have an impact also on the design of an effective NCV, although it remains to be investigated (Fig. 1).

In conclusion, an effective neoantigen prediction pipeline should include: identification of mutations at DNA level, expression from RNA-seq and binding prediction to the MHC of the carrier’s HLA and final modeling of neoantigen quality.

## Conclusions

**Fig. 2:**
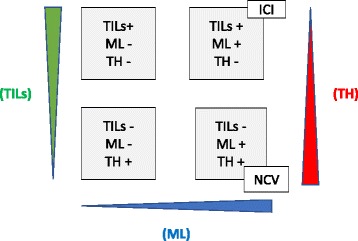
Personalized NCV in the context of current immunotherapy, the three dimensions are defined by tumor infiltrating lymphocytes (TILs), mutational load (ML) and tumor heterogeneity (TH). Patients in the lower right panel (TIL^−^ML^+^TH^+^) may benefit from Neoantigen cancer vaccine (NCV) approach whereas patients in the upper right panel (TILs^+^ML^+^TH^−^) respond more to immune checkpoint inhibitors (ICI)

In the last few years, NCVs have entered the arena of immune therapy consequently raising great expectations due to the initial results in preclinical reports and more recently in clinical studies. It is likely that advances in identifying neoantigens as well as a more in depth understanding of cancer resistance mechanisms [51–57] will extend the range of tumor types that are eligible for NCVs treatment. Based on the preclinical and clinical data, the question put forward is: which is the most suitable population for NCVs in the current context of approved drugs? It is clear that low TH (TH-) but preexisting immunity, as indicated by the presence of TILs (TILs+), as well as high mutational load, defines the most responsive population to ICI (Fig. 2 upper right quadrant). In contrast, the NCVs approach may be more effective in treating cancers with variants represented at a low allele frequency that respond less to ICI. Induction of a larger repertoire of cancer-specific T-cells by adopting the NCVs approach may lead to a better coverage of TH. The combined action of induced cancer-specific CD8 and CD4 T-cells in the periphery by NCVs is likely to result in higher frequency of TILs in patients co-treated with ICI moving a “cold tumor” from the lower right quadrant to the upper right quadrat of “hot tumors” (Fig. 2). In the clinical world, for instance, these features identify a large number of lung cancer patients that do not respond to pembrolizumab in first or second line treatment [58]. Furthermore in the clinical setting, it has been observed that ICI treatment rescues a limited number of neoantigens-specific T-cells that can be expanded in combination with NCVs [37, 38]. However, for both ICI and NCV approaches, a functional HLA presentation machinery is required, as it would be meaningless to treat a patient with ICI if the β2 microglobulin gene is mutated [59]. Induction of an effective T-cell response may be insufficient due to tumor evasion strategies other than PD1 or CTLA-4. A more in-depth knowledge of the tumor microenvironment is therefore required to deliver the right NCVs treatment to the right patient in the best responsive conditions.

## Perspectives

It is clear that a single therapeutic approach will not win the battle against a complex and evolving system such as Cancer. Intrinsic factors such as BRAF mutations are associated with a reduced frequency of TILs, which increase upon a short pharmacological intervention in conjunction with ICI [60]. It is reasonable to expect that similar strategies will be effective with NCV. Therapies against a single target leads in most cases to the selection of genetic variants, which invariably lead to tumor relapses. A similar issue was also observed with ICI using anti-PD1 treatments [61]. In line with this concept tumors relapsing under ICI treatment showed a different mutational landscape with a significant selection of a different spectrum of neoeptitope variants [1]. One possible explanation is the suboptimal response against neoantigens [62]. NCVs promise to be a valuable alternative since they can be tailored to target multiple neoepitopes, thus reducing the risk of immune-evasion due to loss of expression of subsets of neoantigens. Furthermore, with the advancement of NGS technologies and with the increasing sensitivity of liquid biopsies it will be possible in the future to design for the same patient sequential NCVs targeting new neoepitopes selected during tumor evolution.

NCVs represent a new form of precision medicine. Several aspects of the NCV approach require further optimization such as the prediction method for CD8 and CD4 neoantigens or the need of new models for clinical trials. Although technically complex and expensive, it offers important advantages. As stated before it is expected to widen the spectrum of patients responsive to ICI and to synergize with it, for example in cases of relapse to ICI treatment, as reported for the three melanoma patients treated with NCVs followed by ICI [37, 38]. In addition, it may offer a practical advantage to currently untreatable patients approach. For instance, a lung cancer patient with no ALK or ROS translocations and EGFR mutations and with a relatively low expression of PD-L1 and a medium to high neoantigen load would be eligible for the adjuvant NCV approach.

Finally, NCVs pose significant manufacturing, regulatory and marketing issues. The authorization process for a new drug is usually based on expensive large scale randomized clinical trials. This is not feasible with individualized therapies such as NCVs. Pleasingly, this paradigm is changing also thanks to the success of CAR-T therapies where, for example in the case of Tisagenlecelucel, FDA approval was obtained based on the (striking) results of a registration trial involving only 63 patients. Individualized therapies such as CAR-T have also set the ground for very high costs. Are NCVs expected follow the same paradigm? And if so, how sustainable are the increasing costs of personalized therapies in financially “stressed” health systems? These are all important questions that need to be addressed to allow our patients access to innovation.

## Abbreviations

APC: Antigen presenting cells
CTAG1A also known as NY-ESO-1: Cancer-testis antigen
CTAs: Cancer testis antigens
CTLA-4: Cytotoxic T lymphocyte antigen 4
DC: Dendritic cells
FPKM: Fragments Per Kilobase of transcript per Million mapped reads
HBV: Hepatitis B virus
HER2: Epidermal growth factor receptor 2
HLA: Human leukocyte antigen
HPV: Human papillomavirus
ICI: Immune check point inhibitors
MAGE: Melanoma-associated antigen
MART1: Melanoma antigen recognized by T cells
MCC: Merkel cell carcinoma
MHC: Major histocompatibility complex
NCV: Neoantigen cancer vaccine
NGS: Next generation sequencing
NSCLC: Non-small-cell lung cancer
PAP-GMCSF: Granulocyte-macrophage colony-stimulating factor
PD1: Programmed cell death protein 1
PSA: Prostate-specific antigen
RPM: Reads per Million mapped reads
SAGE1: Sarcoma antigen 1
TAAs: Tumor associated antigens
TCR: T cell receptor
TERT: Human telomerase reverse transcriptase
TH: Tumor heterogeneity
TILs: Tumor infiltrating lymphocytes
TSAs: Tumor specific antigens
VCF: Variant calling format
